# DNA polymerase β decrement triggers death of olfactory bulb cells and impairs olfaction in a mouse model of Alzheimer's disease

**DOI:** 10.1111/acel.12541

**Published:** 2016-09-30

**Authors:** Magdalena Misiak, Rebeca Vergara Greeno, Beverly A. Baptiste, Peter Sykora, Dong Liu, Stephanie Cordonnier, Evandro F. Fang, Deborah L. Croteau, Mark P. Mattson, Vilhelm A. Bohr

**Affiliations:** ^1^ Laboratory of Molecular GerontologyNational Institute on Aging Intramural Research ProgramBiomedical Research Center251 Bayview BlvdBaltimoreMD21224USA; ^2^ Laboratory of NeurosciencesNational Institute on Aging Intramural Research ProgramBiomedical Research Center251 Bayview BlvdBaltimoreMD21224USA

**Keywords:** DNA repair, DNA damage, DNA polymerase β, Alzheimer's disease, neurogenesis, olfactory bulb, smell

## Abstract

Alzheimer's disease (AD) involves the progressive degeneration of neurons critical for learning and memory. In addition, patients with AD typically exhibit impaired olfaction associated with neuronal degeneration in the olfactory bulb (OB). Because DNA base excision repair (BER) is reduced in brain cells during normal aging and AD, we determined whether inefficient BER due to reduced DNA polymerase‐β (Polβ) levels renders OB neurons vulnerable to degeneration in the 3xTgAD mouse model of AD. We interrogated OB histopathology and olfactory function in wild‐type and 3xTgAD mice with normal or reduced Polβ levels. Compared to wild‐type control mice, Polβ heterozygous (Polβ^+/−^), and 3xTgAD mice, 3xTgAD/Polβ^+/−^ mice exhibited impaired performance in a buried food test of olfaction. Polβ deficiency did not affect the proliferation of OB neural progenitor cells in the subventricular zone. However, numbers of newly generated neurons were reduced by approximately 25% in Polβ^+/−^ and 3xTgAD mice, and by over 60% in the 3xTgAD/Polβ^+/−^ mice compared to wild‐type control mice. Analyses of DNA damage and apoptosis revealed significantly greater degeneration of OB neurons in 3xTgAD/Polβ^+/−^ mice compared to 3xTgAD mice. Levels of amyloid β‐peptide (Aβ) accumulation in the OB were similar in 3xTgAD and 3xTgAD/Polβ^+/−^ mice, and cultured Polβ‐deficient neurons exhibited increased vulnerability to Aβ‐induced death. Olfactory deficit is an early sign in human AD, but the mechanism is not yet understood. Our findings in a new AD mouse model demonstrate that diminution of BER can endanger OB neurons, and suggest a mechanism underlying early olfactory impairment in AD.

## Introduction

Alzheimer's disease (AD) is characterized by dysfunction and degeneration of neurons in brain regions involved in learning and memory, which is associated with accumulations of aggregated extracellular amyloid β‐peptide (Aβ) and intracellular hyperphosphorylated Tau protein (Mattson, 2004; Crews & Masliah, 2010). Patients with AD also exhibit impaired olfaction, which often occurs early in the disease process (Attems *et al*., 2014); pathology in the olfactory bulb (OB) includes Aβ deposits (Kovacs *et al*., 1999) and neuronal death (Struble & Clark, 1992). Olfactory deficits are evident in patients with mild cognitive impairment and may predict conversion to AD (Attems *et al*., 2015). The mechanisms responsible for the degeneration of OB neurons in AD are unknown, and understanding the cellular and molecular mechanisms may provide new insight into AD pathogenesis and potential early interventions to forestall the disease process.

Interneurons and granule neurons in the olfactory bulb are continuously replaced during adult life from a population of self‐renewing neural progenitor cells located in the subventricular zone (Brann & Firestein, 2014). Newly generated neurons migrate to the OB via the rostral migratory stream and then differentiate into mature neurons that integrate into the OB circuitry. Neurogenesis declines during normal aging (Lazarov *et al*., 2010), and in amyloid precursor protein (APP)‐mutant mice (Haughey *et al*., 2002), which also exhibit impaired olfaction (Guerin *et al*., 2009). It was recently reported that 3xTgAD mice exhibit a deficit in a sexual olfactory preference test (Coronas‐Sámano *et al*., 2014), but the underlying cellular and molecular mechanisms are unknown.

Previous studies of brain tissue samples from human subjects have shown that excessive DNA damage, particularly oxidative modifications, occurs in mild cognitive impairment (MCI) and AD (Wang *et al*., 2006). Unrepaired damage to DNA also occurs during normal aging and may adversely affect genes involved in neuroplasticity (Lu *et al*., 2004). DNA base excision repair (BER), the major mechanism of DNA repair in neurons, is impaired in patients with MCI and AD (Weissman *et al*., 2007). BER corrects DNA lesions through the action of DNA glycosylases that excise damaged bases, AP endonucleases that initiate removal of abasic sites, DNA polymerases that insert the correct base(s), and DNA ligases that reseal the DNA backbone (Sykora *et al*., 2013; Wallace, 2014). The primary polymerase involved in BER is DNA polymerase beta (Polβ). Mice that are null for Polβ die in utero testifying to the importance of this protein. Consistent with a particular vulnerability of neurons to reduced BER, the repair of oxidative DNA damage in neurons is heavily dependent on Polβ (Sykora *et al*., 2015).

Base excision repair activity and Polβ levels decline in parallel in brain cells during aging (Krishna *et al*., 2005; Sykora *et al*., 2015). We recently generated Polβ heterozygous 3xTgAD mice and found that they exhibited degeneration of neurons in the hippocampus and accelerated cognitive deficits that are not evident in 3xTgAD mice (Sykora *et al*., 2015). Interestingly, immature neurons that are newly generated from progenitor cells exhibit greater sensitivity to DNA damage‐induced apoptosis than do neural progenitor cells or mature neurons (Cheng *et al*., 2007). Because newly generated neurons may be particularly sensitive to DNA damage (Cheng *et al*., 2007), and because olfactory neurogenesis is essential for maintenance of optimal olfactory function, we determined whether newly generated OB neurons are vulnerable to diminished BER capacity in a mouse model of AD. We find that Polβ reduction in 3xTgAD mice results in extensive DNA damage and death of newly generated neurons in the OB, and an associated deficit of olfactory function. These findings suggest a role for compromised OB neuron BER in the early loss of OB neurons and consequent olfactory impairment in AD.

## Results

### Polβ heterozygosity elicits an olfactory deficit in 3xTgAD mice

**Figure 1:**
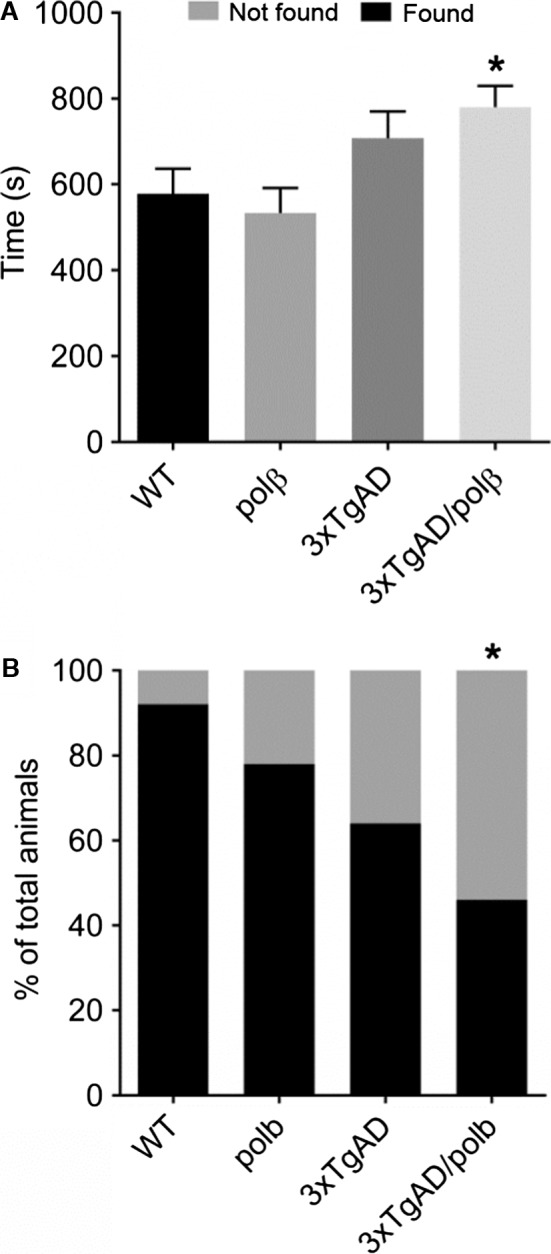
Polβ haploinsufficiency elicits an odor perception deficit in 3xTgAD mice. A and B. Mice of the indicated genotypes were tested in a buried cookie odor perception test. Within the test time of 900 s, the time mice spent finding the buried food was measured and presented as the average time to extract the buried peanut butter cookie (A). The percentage of mice that were successful (found) or not successful (not found) in finding the buried food, within the test time of 900 s. If a mouse could not find the cookie before maximum test time was completed, it got 900 s score (B). n = 8–13 mice/group. Values are the mean and SEM, *P < 0.05 compared to each of the other three groups. ANOVA F(3,44) = 4.029, P value = 0.0129.

To determine whether olfactory function was altered in 3xTgAD mice and/or by reduced Polβ levels, we performed a buried food test on wild‐type, 3xTgAD, Polβ^+/−^, and 3xTgAD/Polβ^+/−^ mice. The rank order of performance (most to least successful in finding the buried cookie) of mice in the four groups was wild‐type > Polβ^+/−^ > 3xTgAD > 3xTgAD/Polβ^+/−^ (Fig. 1). A significantly lower percentage of 3xTgAD/Polβ^+/−^ mice located the buried cookie compared to wild‐type, Polβ^+/−^, and 3xTgAD mice. Indeed, 7 of 13 3xTgAD/Polβ^+/−^ mice did not find a cookie within the 15‐min test period (Fig. 1A). The 3xTgAD/Polβ^+/−^ mice required a significantly longer time to reach the buried cookies (780 s) compared to wild‐type (577 s) and Polβ^+/−^ mice (533 s) (Fig. 1B), indicating impairment in olfactory performance.

### A reduction of Polβ levels results in decreased neurogenesis in the olfactory bulb of 3xTgAD mice

To confirm that Polβ^+/−^ mice do in fact have reduced levels of Polβ in OB cells, we performed immunoblots on OB tissue samples from all four genotypes of mice. The results show that Polβ^+/−^ mice and 3xTgAD Polβ^+/−^ mice have approximately 50% lower amounts of Polβ protein in their OBs compared to wild‐type and 3xTgAD mice (Figure S1). These findings are consistent with a similar magnitude of reduction of Polβ levels in the cerebral cortex of Polβ^+/−^ mice and 3xTgAD Polβ^+/−^ mice (Sykora *et al*., 2015).

**Figure 2:**
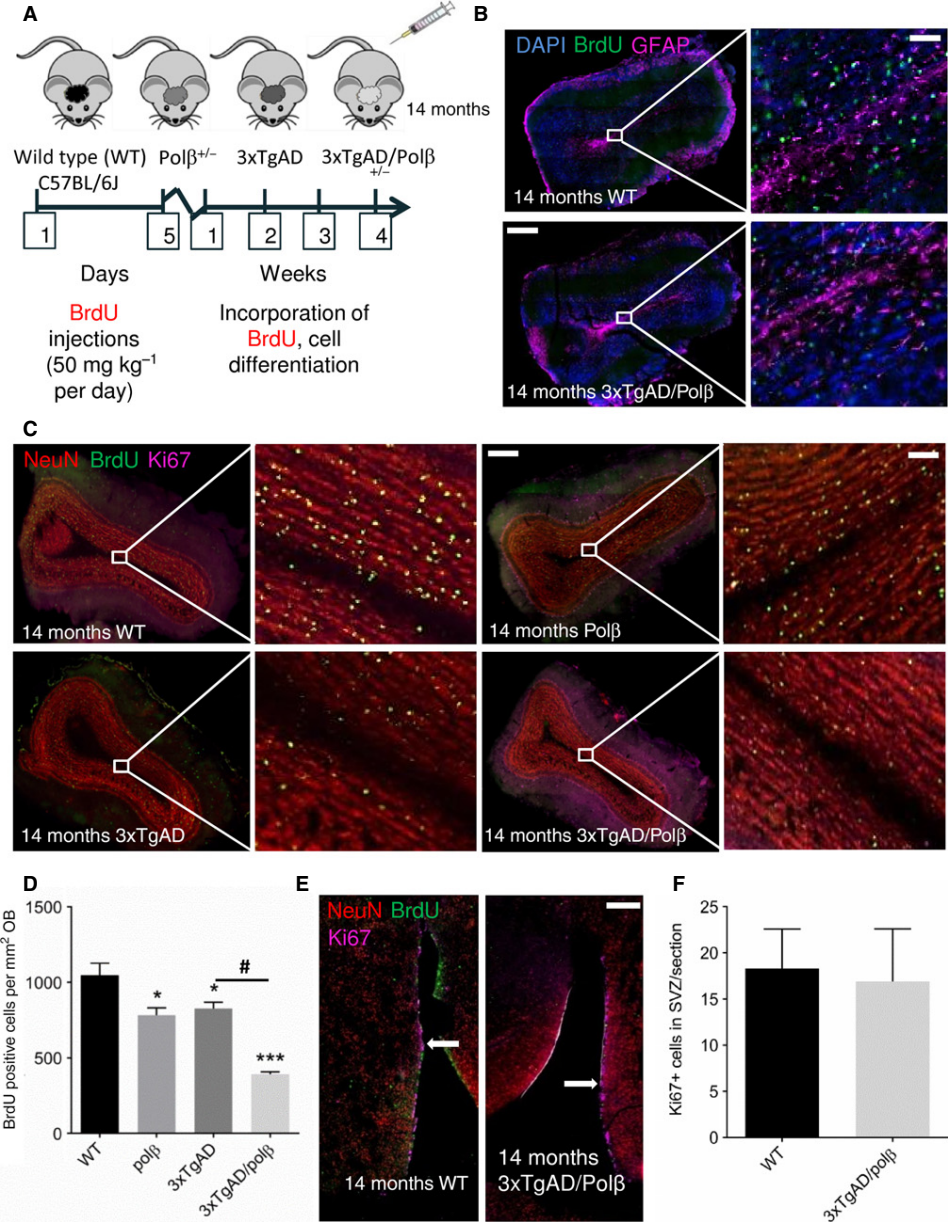
Polβ deficiency exacerbates impaired OB neurogenesis in 3xTgAD mice. A. Experimental design. BrdU was administered once daily for 5 consecutive days, and 4 weeks later, mice were euthanized and brain tissue was analyzed. B. Coronal sections of the OB of 14‐month‐old 3xTgAD and 3xTgAD/Polβ^+/−^ mice stained with DAPI to label nuclear DNA (blue), BrdU to label dividing cells (green), and GFAP antibody to label astrocytes (purple). Note the dearth of BrdU‐labeled cells in the 3xTgAD/Polβ^+/−^ mouse compared to the 3xTgAD mouse. Scale bar = 300 μm and 80 μm. C. Coronal sections of 14‐month‐old mice of the indicated genotypes stained with neuronal marker NeuN (red), BrdU (green), and the cell proliferation marker Ki67 (purple). Scale bar = 300 μm and 100 μm. D. Quantification of BrdU‐labeled cells in the OB (4 weeks after BrdU administration) in mice of the indicated genotypes (n = 4 mice/group). Values are the mean and SEM, *P < 0.05, ***P < 0.0001 compared to the WT or #*P* < 0.001 compared to 3xTgAD group value. ANOVA F(3,12) = 31.46 *P* < 0.0001. E. Coronal brain sections showing the subventricular zone (SVZ) of a WT mouse and a 3xTgAD/Polβ^+/−^ mouse stained with NeuN (red), BrdU (green), and Ki67 (purple). Scale bar = 300 μm. F. Quantification of Ki67‐labeled cells in the SVZ of WT and 3xTgAD/Polβ^+/−^ mice (n = 4 mice/group). Values are the mean and SEM.

Olfactory bulb interneurons and granule cells are derived from stem cells located in the subventricular zone (SVZ) that lines the rostral part of the lateral ventricle (Brazel & Rao, 2004). The production of granule cells for the OB is a continuous process as the SVZ retains proliferative capacity throughout life. To evaluate the consequences of reduced Polβ levels on adult olfactory neurogenesis in an AD mouse model, we administered the DNA precursor BrdU to WT, Polβ^+/−^, 3xTgAD, and 3xTgAD/Polβ^+/−^ mice (14‐month‐old mice littermates) (Fig. 2A). We then waited 4 weeks (a time period during which newly generated cells migrate from the SVZ to the OB and differentiate into neurons). BrdU‐labeled cells in the granule cell layer of the olfactory bulb (OB) were quantified (Fig. 2B–D). There were significant reductions in the numbers of BrdU‐labeled cells in the OBs of 3xTgAD and Polβ^+/−^ mice compared to age‐matched wild‐type mice (Fig. 2D). Numbers of BrdU‐labeled cells in the OBs of 3xTgAD/Polβ ^+/−^ mice were significantly less than in the 3xTgAD or Polβ^+/−^ mice (Fig. 2C,D). To determine whether SVZ stem cell proliferation is altered in 3xTgAD/Polβ^+/−^ mice, we compared numbers of Ki67‐positive cells in the SVZ of 3xTgAD/Polβ^+/−^ mice and age‐matched wild‐type mice and found no difference (Fig. 2E,F). Collectively, these findings suggested that Polβ heterozygosity does not adversely affect SVZ neural stem cell proliferation.

### Polβ heterozygosity exacerbates DNA damage and apoptosis in the OB of 3xTgAD mice

**Figure 3:**
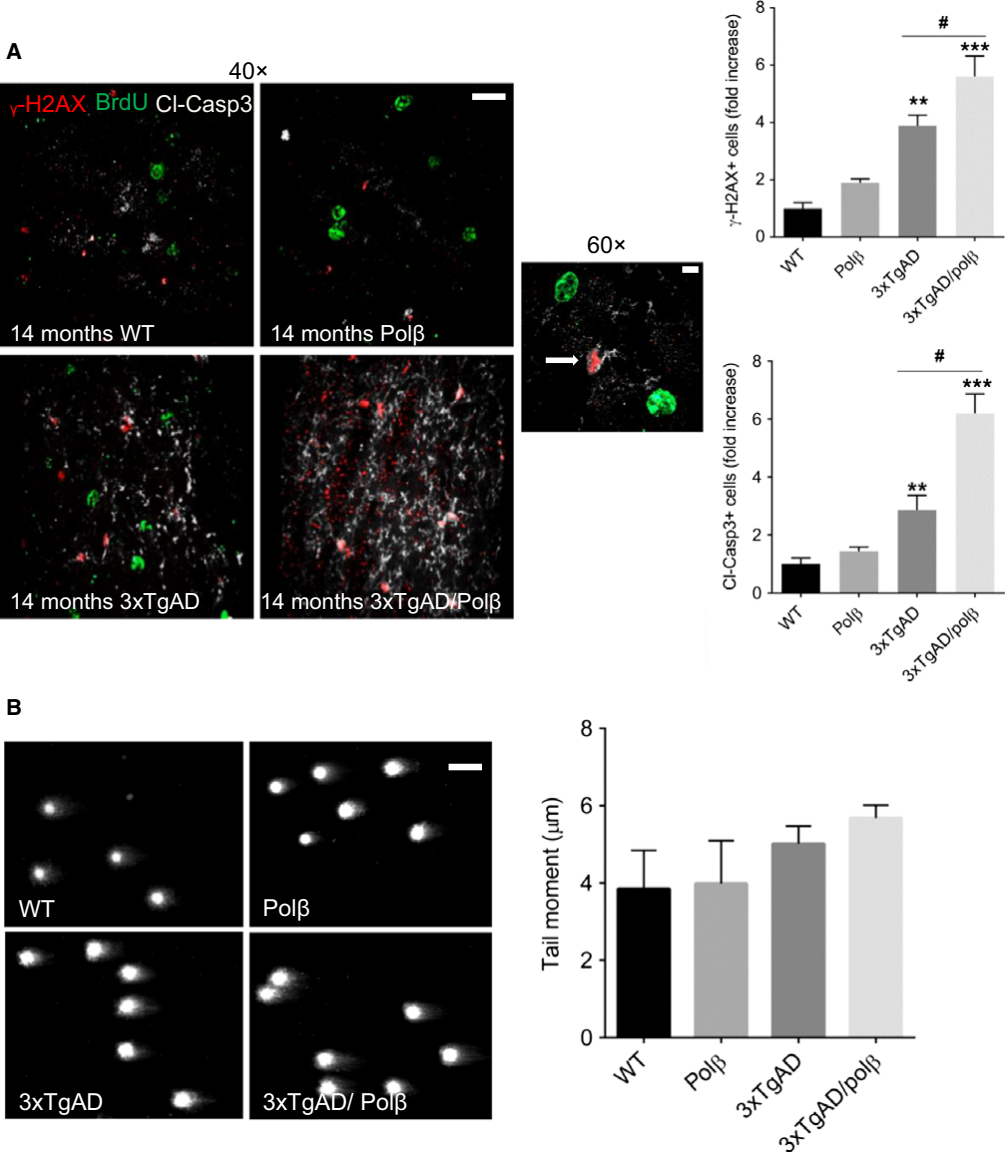
Polβ haploinsufficiency exacerbates DNA damage and apoptosis in OB cells of 3xTgAD mice. A. Images of OB sections of 14‐month‐old mice of the indicated genotypes showing γ‐H2AX immunoreactivity (a marker of DNA double‐strand breaks; red), BrdU (green), and cleaved caspase 3 (a marker of apoptosis; white/gray). Scale bar = 35 μm. The graphs at the right show the results of quantifications of γ‐H2AX immunoreactive foci (upper graph) and cleaved caspase 3 immunoreactivity (lower graph). Values are the mean and SEM (n = 4 mice/group). **P < 0.01, ***P < 0.001 compared to the WT and Polβ values, #*P* < 0.01 compare to 3xTgAD group value. ANOVA F(3,13)=26.64 and F(3,13)=33.79 *P* < 0.001. B. Representative pictures and results of neutral comet analysis of nuclear DNA damage (the length of the olive tail moment) in OB tissue samples.

We next determined the relative amounts of cells with DNA double‐strand breaks (DSBs) in the OB of our mouse models by quantifying γH2AX immunoreactive foci (Fig. 3A). Compared to wild‐type mice, there were significant increases in DSB foci in 3xTgAD (~fourfold) and 3xTgAD/Polβ^+/−^ (~5.5‐fold) mice. In addition, we used the neutral comet assay to quantify nuclear DNA damage in OB tissue and found a trend toward elevated DNA damage in 3xTgAD and 3xTgAD/Polβ^+/−^ mice compared to wild‐type and Polβ^+/−^ mice (Fig. 3B). Because DNA damage can trigger apoptosis, we evaluated levels of cleaved caspase 3 in OBs of mice of each of the four genotypes. The high magnification (60X objective) image in Fig. 3A shows cleaved caspase 3‐positive cells with apoptotic γH2AX‐positive nuclei. 3xTgAD and 3xTgAD/Polβ^+/−^ mice exhibited greater amounts of cleaved caspase 3 compared to wild‐type and Polβ^+/−^ mice (Fig. 3A). The 3xTgAD/Polβ^+/−^ mice had significantly greater amounts of cleaved caspase 3 compared to either 3xTgAD or Polβ^+/−^, indicating that a reduction of Polβ levels exacerbates apoptosis of OB cells in 3xTgAD mice.

### 3xTgAD and 3xTgAD mice with reduced Polβ levels exhibit accumulation of Aβ in OB cells

**Figure 4:**
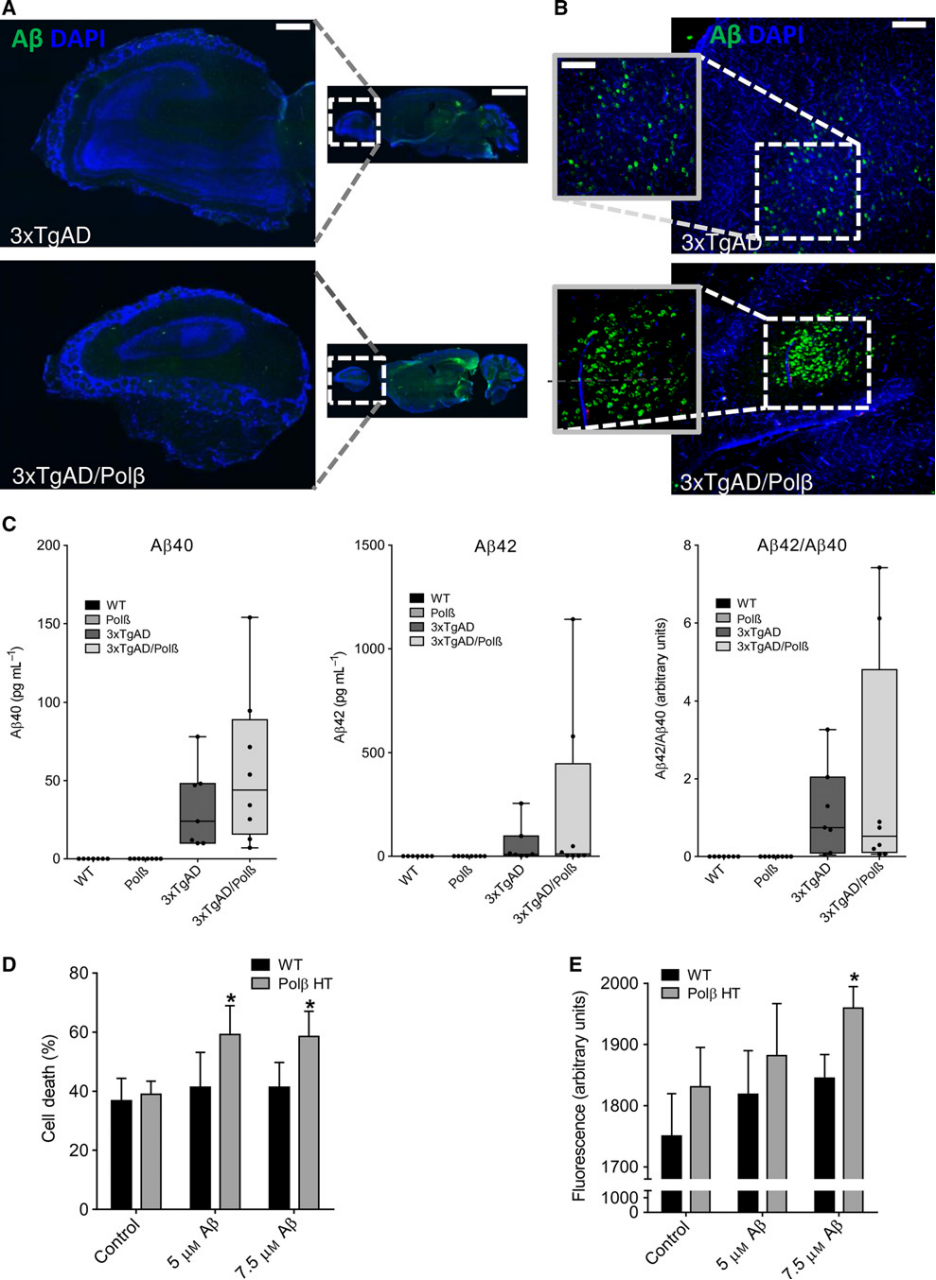
Evidence that Polβ deficiency increases neuronal vulnerability to Aβ‐induced death. A. Images showing Aβ immunoreactivity in sagittal sections of OB in 20‐month‐old 3xTgAD and 3xTgAD/Polβ^+/−^ mice. Scale bar = 300 μm and 3000 μm. B. Images showing Aβ immunoreactivity in sagittal sections of orbitofrontal cortex in 20‐month‐old 3xTgAD and 3xTgAD/Polβ^+/−^ mice. Scale bar = 100 μm and 200 μm. C. Graphs showing results of measurements of levels of Aβ40 and Aβ42, and the Aβ42/Aβ40 ratio in OB tissue samples from mice of the indicated genotypes. Values are the mean, median, and SEM (n = 7 mice per group). Data analyzed by Kruskal–Wallis test showed no significant differences between 3xTgAD and 3xTgAD/Polβ^+/−^ mice. D. Primary cultures of cerebral cortical neurons from wild‐type and Polβ^+/−^ (Polβ ΗΤ) mice were exposed for 24 h to vehicle (control), 5 μM Aβ1‐42, or 7.5 μM Aβ1‐42. Levels of LDH released from the neurons into the culture medium were then quantified as a measure of cell death (see Methods). Values are the mean and SEM of six separate cultures. *P < 0.05 compared to the WT value (t = 2.503, df=19). ANOVA F(5,48) = 2.426 P value = 0.0468. E. Primary cultures of cerebral cortical neurons from wild‐type and Polβ^+/−^ (Polβ ΗΤ) mice were exposed for 24 h to vehicle (control), 5 μM Aβ1‐42, or 7.5 μM Aβ1‐42. Levels of cellular DCF fluorescence were then quantified as a measure of oxidative stress (see Methods). Values are the mean and SEM of six separate cultures. *P < 0.05 compared to the WT value. ANOVA F(5,48) = 2.514 P value = 0.0423.

To examine Aβ accumulation in the OB, we evaluated Aβ immunoreactivity in the OBs brain sections derived from mice of the four genotypes. Levels of Aβ immunoreactivity in the OB were low and not discernably different in 3xTgAD and 3xTg/Polβ^+/−^ mice (Fig. 4A). However, in the orbitofrontal cortex, a cortical target of OB projection neurons, there were significantly more neurons exhibiting intracellular Aβ in 3xTg/Polβ^+/−^ mice than in the 3xTgAD mice (Fig. 4B). We also measured the concentrations of soluble Aβ40 and Aβ42 in OB tissue samples from mice of all four genotypes using an assay that detects human Aβ. As expected, levels of Aβ40 and Aβ42 were very low in nontransgenic mice (wild‐type and Polβ^+/−^) compared to 3xTgAD mice (Fig. 4C). There were no significant differences in levels of Aβ40 and Aβ42, or the Aβ42/Aβ40 ratio between 3xTgAD mice and 3xTg/Polβ^+/−^ mice (Fig. 4C).

It has been reported that Aβ can be neurotoxic and can increase the vulnerability of neurons to excitotoxic, metabolic, and oxidative stress (Mattson *et al*., 1992; Suberbielle *et al*., 2013). To determine whether Polβ deficiency increased the vulnerability of neurons to Aβ, we cultured primary cerebral cortical neurons from wild‐type and Polβ^+/−^ mouse embryos and exposed the neurons to two concentrations of Aβ1‐42 (Fig. 4D). Cell death was quantified by measuring lactate dehydrogenase (LDH) release into culture medium. Cell death was significantly greater in Polβ^+/−^ neurons compared to WT neurons after exposure to Aβ1‐42 (Fig. 4D). At the highest Aβ concentration, DCF fluorescence, an indicator of reactive oxygen species, was more prominent in Polβ^+/−^ neurons than in WT neurons exposed to Aβ1‐42 (Fig. 4E). Thus, a reduction in Polβ levels sensitizes neurons to Aβ‐induced ROS production and cell death.

### Polβ deficiency leads to failed development of olfactory bulb in embryos and to a metabolic crisis in newly generated neurons

**Figure 5:**
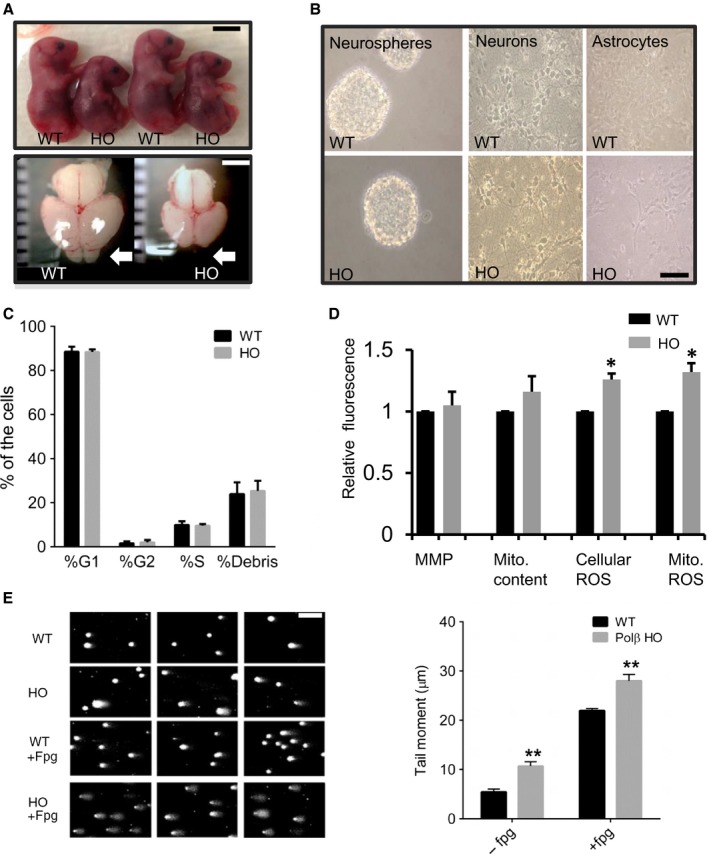
Polβ deficiency impairs olfactory bulb formation and causes DNA damage in newly generated neurons, while not adversely affecting NPC proliferation or mitochondrial function. A. Images showing WT and Polβ‐deficient (HO) embryos and whole brain (E17). Polβ‐deficient embryos are smaller than WT embryos. Scale bar = 1 cm. The brains of Polβ‐deficient embryos are smaller than their WT counterparts and do not develop a discernable olfactory bulb (arrow). Scale bar = 2 mm. B. Images showing neurospheres, and neurons and astrocytes differentiated from neurospheres, in cultures established from WT and Polβ^−/−^ embryos. Scale bar = 100 μm. C. Results of flow cytometry cell cycle analysis of NPCs from WT and Polβ^−/−^ embryos. D. Results of flow cytometry‐based measurements of the indicated parameters: MMP, mitochondrial membrane potential; Mito. content, mitochondrial DNA content; cellular reactive oxygen species (ROS); and mitochondrial ROS. E. Results of alkali comet assay with formamidopyrimidine DNA glycosylase (Fpg) enzymatic digestion. Comet assay analysis revealed accumulation of oxidative DNA damage in Polβ‐deficient neurons. n ≥ 5. *** *P* < 0.001. ANOVA; F(3,19) = 166.2; *P* < 0.0001.

It was previously reported that Polβ^−/−^ mice die in utero or upon birth, that the embryos are smaller, and that they exhibit extensive neurodegeneration (Sugo *et al*., 2000); however, this phenotype has not been fully explored. When we examined the brains of the Polβ^−/−^ embryos (E17), we found a severe deficiency of the development of their olfactory bulbs and that their brains were smaller than those of their wild‐type littermates (Fig. 5A). To validate the in vivo results, showing a deficiency in adult neurogenesis in 3xTgAD mice with reduced Pol β level, we also evaluated neural primary cells. To study the consequences of Polβ deficiency on the proliferation and differentiation of neural progenitor cells (NPCs), we propagated WT and Polβ^−/−^ embryonic neural progenitor cells in neurosphere cultures established from E13.5 embryos (Fig. 5B). We first determined whether Polβ deficiency affects NPC proliferation by dissociating cells from neurospheres and performing flow cytometry analysis of their cell cycle status. There were no significant differences between the percentages of WT and Polβ^−/−^ NPCs in any of the three phases of the cell cycle (G1, G2, and S) (Fig. 5C). To assess the differentiation potential of the NPCs, the cells in neurospheres were dissociated and plated on an adherent substrate under conditions that result in the differentiation of either neurons or astrocytes (see Methods). Microscopy analysis showed that both the Polβ^−/−^ and WT NPCs were similarly capable of differentiating into neurons and astrocytes, when placed in Neurobasal media or serum enhanced DMEM, respectively (Fig. 5B).

Recent findings suggest that nuclear DNA damage can trigger signaling pathways that affect mitochondrial function (Fang *et al*., 2014; Fang *et al*., 2016). Moreover, Sykora *et al*. (2015) found severe energetic dysfunction in the (3xTgAD/Polβ^+/−^) AD mouse model. We therefore evaluated parameters of mitochondrial function and oxidative stress in WT and Polβ^−/−^ NPCs and found no significant differences in mitochondrial membrane potential. However, there was a significant increase in cellular and mitochondrial ROS in the Polβ^−/−^ NPCs (Fig. 5D).

**Figure 6:**
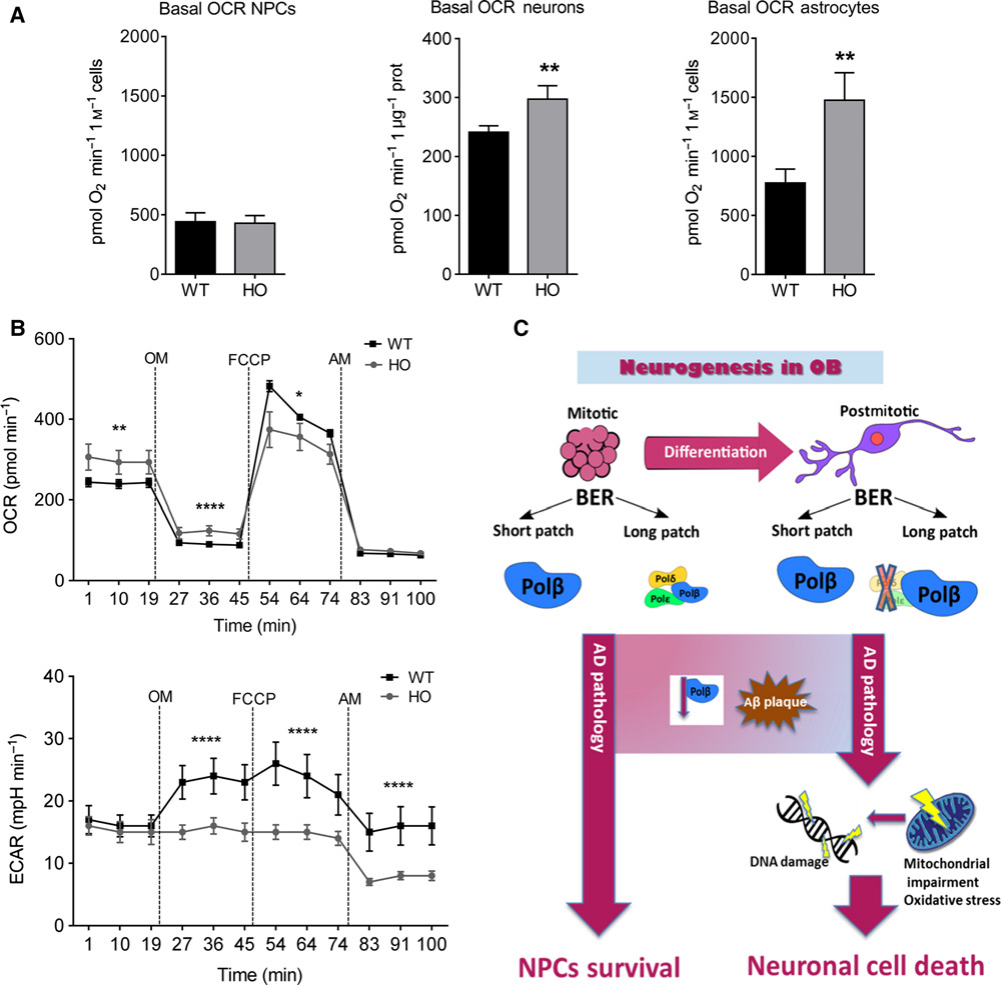
Polβ deficiency in differentiated cells alters mitochondrial respiration. A. Basal oxygen consumption rates (OCR) in neural progenitor cells (NPCs), and neurons and astrocytes differentiated from NPCs from WT and Polβ‐deficient mouse embryos. n = 4 separate experiments for neurons, and 7 separate experiments for NPCs and astrocytes. *P < 0.05, **P < 0.05. B. Results of Seahorse analysis of mitochondrial function in WT and Polβ‐deficient neurons. OCRs and ECARs were measured under basal condition first (9XF Seahorse Bioscience machine), followed by sequential addition of oligomycin (OM), FCCP, and antimycin A (AM). All the OCR and extracellular acidification rate (ECAR) values presented were normalized to protein content. Polβ‐deficient neurons respire on higher rates and exhibit lower reserve capacity when compared to WT neurons, suggesting a deregulation of energy metabolism. Moreover, Polβ‐deficient neurons were unable to utilize energy from other sources after blocking respiration with oligomycin. n = 4 separate experiments. *P < 0.05, ** *P* < 0.01, ***P < 0.001, ****P < 0.0001. C. Schematic diagram showing how Polβ may protect newly generated olfactory neurons against Aβ toxicity. Long patch BER in mitotic neural progenitor cells (NCPs) depends on Polβ and other replicative polymerases, whereas in differentiated postmitotic neurons, BER depends mainly on Polβ. In AD, neurons are exposed to potentially toxic forms of Aβ. Whereas Polβ may normally protect neurons against AD, reduced levels of Polβ during aging and AD endanger neurons, rendering them vulnerable to Aβ, and metabolic and oxidative cellular stress.

We next assessed nuclear DNA damage in neurons that had been differentiated from WT and Polβ^−/−^ NPCs using a comet assay performed in the absence or presence of Fpg, an endonuclease that specifically incises oxidatively modified bases. Polβ^−/−^ neurons exhibited significantly more DNA damage, indicated by a greater olive tail moment, in both the absence and presence of Fpg (Fig. 5E). The shift from anaerobic glycolysis to oxidative phosphorylation is critical to support the high energy demands of differentiating cells, and it was previously reported that newly generated neurons exhibit increased vulnerability to DNA damage (Cheng *et al*., 2007). We therefore evaluated mitochondrial oxygen consumption in NPCs, and neurons and astrocytes differentiated from WT and Polβ null embryos (Fig. 6A). There were no significant differences in basal oxygen consumption rate (OCR) between WT and Polβ^−/−^ NPCs. In contrast, there the OCRs of Polβ^−/−^ neurons and astrocytes were significantly greater than their WT counterparts (Fig. 6A). Additionally, we found that neurons lacking Polβ exhibited significantly lower reserve capacity when compared with WT neurons and were unable to shift from oxidative phosphorylation to anaerobic glycolysis or pentose–phosphate pathway, when challenged with oligomycin, an ATP synthase inhibitor (Fig. 6B). Collectively, these findings suggest that a deficit in DNA repair does not adversely affect NPCs, but results in mitochondrial dysfunction, oxidative stress, DNA damage, and cell death in newly generated neurons (Fig. 6C).

## Discussion

We characterized 3xTgAD/Polβ^+/−^ mice at the molecular, cellular, and behavioral levels and found that Polβ heterozygosity exacerbates olfactory dysfunction and further promotes degeneration of newly generated OB cells in 3xTgAD mice. We labeled SVZ stem cells and their OB progeny and found that, whereas Polβ heterozygosity had no effect on numbers of BrdU‐labeled cells in the SVZ of 3xTgAD mice, it caused a dramatic reduction in BrdU‐labeled cells in the OB. These findings suggest that newly generated neural cells arising from SVZ stem cells in 3xTgAD/Polβ^+/−^ mice die as they migrate from the SVZ to the OB and/or after they are differentiating into neurons in the OB. It was previously reported that newly generated embryonic cortical neurons are much more vulnerable to DNA damage‐induced apoptosis than are NPCs or mature differentiated neurons (Cheng *et al*., 2007). Because we found that OB cells in 3xTgAD/Polβ^+/−^ mice exhibit greatly increased γ‐H2AX immunoreactivity (a marker of DNA double‐strand breaks) and cleaved caspase 3 immunoreactivity (a marker of apoptosis), it appears that many newly generated neurons in those mice die within the OB.

Levels of cell‐associated Aβ immunoreactivity are increased in some brain regions of 3xTgAD/Polβ^+/−^ mice compared to 3xTgAD mice. These include the hippocampus and amygdala (Sykora *et al*., 2015) and the orbitofrontal cortex (present study). However, we found that levels of Aβ immunoreactivity in the OB were similar in 3xTgAD/Polβ^+/−^ and 3xTgAD mice. Similarly, there was no significant difference in the amount of Aβ42 or Aβ40 in the OBs of 3xTgAD and 3xTgAD/Polβ^+/−^ mice. These findings suggest that Polβ heterozygosity may render OB cells vulnerable to Aβ toxicity. In support of the latter possibility, we found that cultured embryonic Polβ^+/−^ neurons were more vulnerable to being killed by Aβ1‐42 compared to wild‐type neurons. In addition, whereas the proliferation and mitochondrial function of NPCs was unaffected by Polβ deficiency, mitochondrial function was compromised in Polβ‐deficient newly generated neurons. Neurons may be particularly vulnerable to Aβ because they are highly excitable cells with a high energy demand, and Aβ can impair the function of membrane ion‐motive ATPases, and glucose and glutamate transporters (Mattson, 2004). As a consequence, Aβ renders neurons vulnerable to excitotoxicity, a mechanism of neuronal degeneration implicated in AD (Mattson *et al*., 1992;). Altogether, data from 3xTgAD mice and cultures cells exposed to Aβ suggest that a reduction in Polβ levels may not adversely affect the proliferation of SVZ NPCs, but does render newly generated OB neurons vulnerable to apoptosis.

Because Polβ expression is known to decrease in brain cells during normal aging and more so in AD (Cabelof *et al*., 2002; Weissman *et al*., 2007), our findings suggest a role for an age‐related decline in Polβ and BER in rendering newly generated OB neurons vulnerable to AD. Previous studies have documented impaired olfactory function in several mouse models of AD. In APP‐mutant transgenic mice, olfactory perception deficits were correlated with the accumulation of nonfibrillar Aβ deposition in the OB (Wesson *et al*., 2010). Another study showed that expression of mutant APP in olfactory sensory neurons disrupts their connectivity and reduces odor‐induced OB cell gene expression and olfactory acuity (Cao *et al*., 2012). It was also reported that olfactory sensory neurons exhibit evidence of apoptosis (cleaved caspase 3 immunoreactivity in mice in which mutant human APP is expressed in those neurons (Cheng *et al*., 2013). The latter study further demonstrated that the olfactory circuit degeneration and associated functional deficits were completely reversed within 1 month of cessation of expression of the mutant APP. None of the previous studies examined the possible roles of DNA damage in the olfactory pathology and associated functional deficits. Our findings suggest a key role for Polβ and BER in protecting newly generated OB neurons against Aβ toxicity.

Accumulating evidence points to a prominent role for impaired DNA repair in the pathogenesis of AD and related neurodegenerative disorders (see Madabhushi *et al*., (2014) and Fang *et al*., 2016 for review). Aging, which is the major risk factor for late‐onset AD, is associated with the accumulation of DNA damage in brain cells (Lu *et al*., 2004), reduced BER activity (Krishna *et al*., 2005), and a reduction in Polβ levels and activity (Cabelof *et al*., 2002; Sykora *et al*., 2015). Premature aging syndromes such as Cockayne syndrome and Werner syndrome are caused by mutations in DNA repair‐related enzymes and exhibit neurodegenerative phenotypes (Kyng & Bohr, 2005). In addition, patients with Down syndrome, who always develop AD‐like neuropathology and cognitive deficits, exhibit reduced levels of Polβ and olfactory deficits (Patterson & Cabelof, 2012). Moreover, mice with reduced DNA repair capability as a result of genetic deletion of BER proteins (OGG1, NEIL1, Xrcc 1, and Polβ) exhibit increased neuronal vulnerability to metabolic stress (Liu *et al*., 2011; Canugovi *et al*., 2012; Ghosh *et al*., 2015), and metabolic stress is implicated in neuronal dysfunction and death in AD (Mattson *et al*., 2008).

Our findings suggest that reduced DNA repair capacity can render newly generated olfactory neurons vulnerable to degeneration in the face of relatively low amounts of accumulating Aβ that do not otherwise cause degeneration of olfactory cells, nor olfactory deficits, in the 3xTgAD mouse model. We also found that Polβ^+/−^ neurons were more vulnerable to Aβ‐induced cytotoxicity and mitochondrial dysfunction compared to wild‐type neurons (Fig. 4D and E), consistent with the possibility that reduced Polβ levels increase the vulnerability of newly generated olfactory neurons to Aβ in 3xTgAD/Polβ^+/−^ mice. Importantly, the approximately 50% reduction of Polβ levels in the Polβ^+/−^ mice is similar to the reduction of Polβ levels that occurs in the brain during normal aging (Cabelof *et al*., 2002). Because age is the major risk factor for olfactory deficits and AD, our findings suggest that Polβ levels may determine whether or not olfactory neuronal pathology and associated functional deficits occur in individuals during aging and in AD. Interventions that can enhance BER, such as synaptic activity and exercise (Yang *et al*., 2010; Yang *et al*., 2014), may protect the olfactory system (and other neuronal networks) against age‐ and AD‐related dysfunction and degeneration.

## Experimental procedures

### Animals

Mice were generated and maintained at the National Institute on Aging Intramural Research Program facility in Baltimore, MD. Mice were maintained on a standard NIH diet and a 12‐h light/dark cycle. Mice were group housed and had ad libitum access to food and water. The original 3xTgAD line (Oddo *et al*., 2003) was backcrossed to C57BL/6 mice for eight generations, and their Aβ and pTau pathologies were characterized (Liu *et al*., 2010). The generation, genotyping, and characterization of Polβ^+/−^ mice were described previously (Sobol *et al*., 1996; Cabelof *et al*., 2003). Polβ^+/−^ mice were provided by A. R. Heydari at Wayne State University, and the Polβ^+/−^ colony was maintained by breeding wild‐type with Polβ^+/−^ mice. Polβ^+/−^ mice were crossed with 3xTgAD mice, and 3xTgAD/Polβ^+/−^ and 3xTgAD/Polβ^+/−^ littermates were used for the experiments. Experiments were performed on 14‐month‐old male (for behavioral tests and immunohistochemical analyses of the OB) or female (for analysis of neurogenesis) mice. All animal procedures were approved by the National Institute on Aging Animal Care and Use Committee and complied with NIH guidelines.

### Olfactory test

The procedure for the buried food test has been described previously (Yang & Crawley, 2009). Several days prior to testing, we familiarized all mice with peanut butter cookies and also tested the consumption of this new food between the groups; mice of all genotypes consumed the cookies equally well. Mice were fasted for 16 h and then placed in a cage containing bedding that was 7.5 cm in depth with three morsels of food (a small piece of peanut butter flavored cereal; Nestle, Columbia, MD). During the test, each mouse was given a 15‐min period to find the buried food. All tests were video‐recorded and used later to establish the latency for the mouse to extract the buried food. We also measured the total time each mouse spent in exploring the entire cage area and found that activity levels were similar in all mouse groups. The person performing the test and analyzing the data was blinded as to the genotype of the mice.

### Preparation of brain tissue sections

Mice were anesthetized by isoflurane inhalation and perfused intracardially with PBS followed by 4% paraformaldehyde in PBS. Mice were decapitated, and brains were immediately removed and placed in 4% paraformaldehyde in PBS for 48 h, followed by equilibration in 30% sucrose. Tissue was sectioned coronally (40 μm) on a freezing microtome (Thermo Fisher Scientific, MD) and stored at −20 °C in tissue cryoprotectant solution.

### BrdU injections, immunohistochemistry, and evaluation of neurogenesis

These methods were similar to those described previously (Kobilo *et al*., 2011). Briefly, mice received single intraperitoneal doses of 50 mg g^−1^ body weight BrdU (Sigma‐Aldrich) once daily for 5 consecutive days. Mice were euthanized, and brains removed and processed for immunohistochemistry 4 weeks after the last BrdU injection. A 1:6 series of equidistant (240 μm between sections) free‐floating 40‐μm coronal sections through the entire rostro‐caudal extent of the olfactory bulb were used. After antigen retrieval, thorough washing and blocking, sections were incubated with rat anti‐BrdU (1:100, Accurate Chemical Co., Westbury NY), mouse anti‐NeuN (1:100, Millipore) and/or rabbit anti‐GFAP (1:500, EnCor Biotechnology, FL) and/or rabbit anti‐Ki67 (1:250, Abcam, MA) for 72 h at 4 °C. After several rinses, sections were co‐incubated with donkey anti‐rat Alexa Fluor 488 (1:250, Molecular Probes, Carlsbad, CA), donkey anti‐mouse Alexa Fluor 568 (1:250, Jackson ImmunoResearch, PA), and donkey anti‐rabbit Cy5 (1:250, Jackson ImmunoResearch, PA) for 3 h at room temperature. Sections were counterstained with DNA‐binding dye DAPI to label all cell nuclei. Images were captured using an Axiovert 200M Zeiss microscope using a 10X objective and AxioVision 4.8.3.0 Mosaics software.

### In situ evaluations of DNA damage and apoptosis

These methods were similar to those described previously (Sykora *et al*., 2015). Briefly, sections of olfactory bulb were immunostained with a mouse monoclonal antibody against phosphorylated γH2AX (Ser139) conjugated to Alexa Fluor^®^ 488 (Cell Signaling Technology, MA) to evaluate DNA double‐strand breaks, and with a rabbit antibody against cleaved caspase 3 (Cell Signaling Technology, MA) to identify apoptotic cells. Immunostained sections were mounted in Vectashield hard set with DAPI and analyzed under an Axiovert 200M Zeiss microscope using 40X and 60X objective lenses. Nuclear foci of γH2AX and cellular cleaved caspase 3 immunoreactivity were quantified in three random areas on at least three sections of OB per mouse, and data are presented as fold of increase compared to WT.

### Amyloid deposition

We detected Aβ using the same tissue preparation as described above and an antibody against amyloid β (#2454, Cell Signaling Technology, MA). The Aβ antibody was raised against N‐terminal residues (672–713 (Aβ42) and 672–711 (Aβ40)) and hence is more specific for these fragments but can also detect other Aβ fragments (such as Aβ‐37, Aβ‐38, and Aβ‐39) and to a lesser extent the N‐terminal of full‐length APP. Brain tissue ≥18 months was investigated from either 3xTgAD or 3xTg/Polβ mice, and WT and Polβ mice do not express human APP or derivatives and were consequently omitted from this analysis. At least three mice from each group were assessed.

### Quantification of soluble Aβ in OB tissue samples

Analysis of Aβ42 and Aβ40 was performed using a MSD Multi‐Spot 96‐Well Plate, Aβ Peptide Panel 1 Kit (V‐Plex, Meso Scale Diagnostics, LLC) according to the manufacturer's protocol. Briefly, blocking solution was added to wells, and 1 h later, the wells were washed three times with buffer solution and detection antibody was added, followed by 100 μg of OB tissue homogenate. Plates were incubated for 2 h at room temperature, washed three times with buffer, 2x Read Buffer was added, and plates were read on a MSD QuickPlex SQ 120 instrument.

### Comet analysis of DNA damage

Brain regions were dissected and homogenized in phosphate‐buffered saline (PBS) on ice. The homogenate was passed through a 0.45‐μm filter to catch any remaining tissue fragments. The extracts were then centrifuged at 800 × *g* for 10 min at 4^◦^C. The supernatant was removed, and the nuclear pellet was weighed, suspended in PBS, and used for the comet assay as described previously (Yang *et al*., 2014). The nuclei were added to melted agarose at a ratio of 4:1 (v/v). Agarose cell suspension was dotted onto the hydrophilic side of a GelBond film (Lonza). The nuclei were lysed overnight at 4^◦^C using a standard neutral comet protocol. Nuclear DNA was electrophoresed at 25 V for 30 min at 4^◦^C and stained with SYBR Gold (Life Technologies, MD). Nuclear comets were imaged using a fluorescence microscope and analyzed using comet analysis software. Samples from mice in all four genotype groups were run together on a single film to enable direct comparisons among groups. At least 50 comet images were acquired and analyzed for each group. For assessing DNA damage in neuronal cells, we performed alkali comet assay on slides, where cells were additionally exposed to formamidopyrimidine DNA glycosylase (Fpg) diluted in FPG FLARE reaction buffer (1x FLARE, 1x BSA, Trevigen, MD) or buffer alone for 1 h at 37 °C. Slides were then incubated in alkali solution (pH 12.1) for 30 min and placed in electrophoresis chamber filled with prechilled 1x TBE buffer and run for 30 min at 35 V. The DNA was then stained with ethidium bromide and viewed on a Zeiss Axiovert 200 M fluorescent microscope (Zeiss, Thornwood NY). The analysis of the comet tail length was performed using Komet 5.5 software (Kinetic Imaging, Durham, NC).

### Culture of neurospheres, and astrocytes and neurons derived therefrom

Neural progenitor cells (NPCs) from the cerebral cortex of embryonic mice were propagated as free‐floating aggregates called neurospheres as described previously (Cheng *et al*., 2010). In brief, the dorsal telencephalon from E14.5 mouse embryos was isolated, and cells were mechanically dissociated and seeded at a density of 200,000 cells ml^−1^ in a T75 flask containing DMEM/Ham's F‐12 medium, supplemented with B27 (1:50; Invitrogen, CA), 20 ng ml^−1^ epidermal growth factor, and 20 ng ml^−1^ fibroblast growth factor 2 (R & D Systems, MN). After 7 days in culture, cells were dissociated using the NeuroCult cell dissociation kit (StemCell Technologies, BC, Canada) and used for experiments or for differentiation into astrocytes and neurons. Dissociated neurospheres were centrifuged at 300 *g* for 5 min. The single cell suspensions were resuspended in Dulbecco's modified Eagle's medium (DMEM; Thermo Fisher Scientific, NY) supplemented with 16% (v/v) fetal calf serum (Thermo Fisher Scientific, NY), 50 U ml^−1^ penicillin, 50 μg ml^−1^ streptomycin, 0.25 μg ml^−1^ amphotericin B (Fungizone), and 2 mM L‐glutamine (Glutamax) and plated onto poly‐L‐ornithine‐coated (PLO, Sigma‐Aldrich, MO) culture dishes at a density of approximately 1 million cells per cm^2^. Astrocyte cultures were grown at 37 °C in a humidified atmosphere of 95% air/5% CO_2_. Subconfluent astrocytes were incubated in Neurobasal medium (NBM) supplemented with 0.2% (v/v) B27, penicillin, streptomycin, Fungizone, and L‐glutamine for 48 h and subsequently used for experiments. For neuronal differentiation, dissociated neurosphere cells were resuspended in Neurobasal medium supplemented with 2% (v/v) B27, 50 U ml^−1^ penicillin, 50 μg ml^−1^ streptomycin, 2 mM L‐glutamine (Glutamax), and 10% (v/v) fetal bovine serum and plated onto PLO‐coated culture dishes at a density of approximately 200,000 cells per cm^2^. Within 6–10 h of cell plating, the culture medium was replaced with serum‐free Neurobasal medium with B27 supplement. This procedure typically yielded neuron‐enriched cultures containing > 90% neurons and < 10% glial cells, mostly astrocytes. Cortical neuronal cultures for Aβ toxicity assays were prepared from WT and Polβ^+/−^ embryos (E16‐17) and plated directly on PLO‐coated culture dishes and cultured in serum‐free Neurobasal medium with B27 supplement for 7 days, followed by 24 h in Neurobasal medium without B27 supplement for treatment with Aβ 1‐42.

### Analyses of mitochondrial function

Mitochondrial membrane potential (MMP), mitochondrial volume, mitochondrial ROS levels, and whole‐cell ROS levels were measured using a BD Accuri^™^ C6 flow cytometer. Briefly, cells were harvested, washed with PBS, and resuspended in DME without phenol indicator (Invitrogen). The cells were then incubated with the following fluorescent probes (all from Life Technologies, MD): TMRM (40 nM for 15 min) to measure relative MMP; MitoTracker Green (50 nm for 30 min) to determine relative mitochondrial volume; dihydroethidium (DHE, 3 μM for 30 min) to detect cellular ROS; and mitoSOX (3 μM for 30 min) for mitochondrial ROS (principally superoxide). Fluorescence intensity/cell was determined in 10,000 cells/culture by flow cytometry (Fang *et al*., 2014). Data were analyzed using FCS Express 4 software. Oxygen consumption and extracellular acidification rate measurements were performed using the Seahorse XF‐24 instrument (Seahorse Biosciences, MA). Cells were seeded into Seahorse tissue culture plates, and 16 h later, the culture medium was changed to unbuffered XF assay medium at pH 7.4 (Seahorse Biosciences, MA), supplemented with 25 mM glucose (Sigma‐Aldrich), 1 mM sodium pyruvate, and 1 mM Glutamax (Invitrogen, CA). Cells were incubated for 1 h at 37 °C at ambient O_2_ and CO_2_ concentration before measurements were taken. Respiration was measured in four blocks of 3 min block^−1^. The first block measured the basal respiration rate. Next, oligomycin (EMD) was added to inhibit complex V and the second block was measured. Then, FCCP (Sigma‐Aldrich) was added to uncouple respiration and the third block was measured. Finally, antimycin A (Sigma‐Aldrich, MO) was added to inhibit complex 3 and the last measurements were performed. All compound concentrations used had been optimized for the cell types being analyzed. Immediately after finishing the measurements, cells were trypsinized and counted using a Coulter counter (Beckman Coulter) or protein content was measured, and respiration was normalized to protein concentration or cell number.

### Lactate dehydrogenase (LDH) activity assay

To estimate cell death, the level of lactate dehydrogenase (LDH) released from damaged cells into culture media was measured by detecting the enzyme activity using a commercially available kit (Life Science, Roche, IN) at 24 h after treatment with 5 and 7.7 μM Aβ1‐42. A colorimetric assay was applied. The absorbance was measured at a wavelength of 490 nm. Data were normalized to LDH activity released from Triton X‐100‐treated control cells (set 100% of cell death).

### Intracellular reactive oxygen species detection

Primary neurons were cultured in 96‐well plates at a density of 5x10^4^ cells/well. After treatment with or without 5 and 7.7 μM Aβ 1–42 for 24 h, cells were loaded with 10 μM H2DCFDA, a precursor of 2,7‐dichlorofluorescein diacetate (DCF) diluted in PBS buffer and incubated for 45 min at 37 °C. The fluorescence intensity of DCF after excitation of the samples at a wavelength of 485 nm was measured (Thermo Fisher Scientific, MD).

### Statistics

Comparison between two groups was analyzed by parametric Student's *t*‐test with Welch's correction. One‐way ANOVA with Tukey's multiple comparison testing was used to determine statistical significance among multiple groups. If the data were not normally distributed, nonparametric Mann–Whitney *U*‐test or Kruskal–Wallis test was applied. Nonparametric chi‐squared test was used to prove null hypothesis in olfactory test. Prism analysis software (PRISM 6, GraphPad, La Jolla, CA, USA) was used for all analyses.

## Conflict of interest

The authors declare no competing financial interests.

## Funding

This work was supported by the Intramural Research Program of the National Institute on Aging.

## Supporting information


**Data S1** Immunoblotting

**Fig. S1** Polβ proteins level in Olfactory Bulbs.
