# Supplementary material for: DNA polymerase β decrement triggers death of olfactory bulb cells and impairs olfaction in a mouse model of Alzheimer's disease
Source: Aging Cell. 2016 Sep 30;16(1):162–72. doi: 10.1111/acel.12541 (PMC5242308; doi:10.1111/acel.12541)
Supplement: Supplementary file 1 — Data S1 Immunoblotting [file ACEL-16-162-s001.docx]

**Supplementary methods:**

**Immunoblotting**

Olfactory bulbs were isolated from mice from all genotypes, lysed with 1xRIPA lysis buffer (Cell Signaling Technology), sonicate and extracts were prepared after centrifugation at 13000g, 4 ^0^C for 10 min. Proteins were resolved in 4-15% PROTEAN TGX gels (BioRad), electroblotted to nitrocellulose membrane, and visualized using antibodies against DNA Polymerase β (1:1000, Ab26343, abcam, MA), actin (sc-1616, Santa Cruz).

**Suplementary figure legend:**

**Supplementary Figure 1**. **Polβ deficiency in differentiated cells targets mitochondrial respiration. A.** Western Blots showing relative levels of Polβ and actin proteins in OBs from mice of the indicated genotypes. 7 mice for WT, 6 mice for Polβ^+/-^ and 9 mice for 3xTgAD, and 3xTgAD/Polβ^+/-^ were used. As a positive and negative control we used protein extracts from Polβ^+/+^ and Polβ^-/-^ cell lines. **B.** Graph showing results of quantification of Polβ levels normalized to actin levels, in OBs of mice of the indicated genotypes. Values are the mean and SEM. *p<0.05.
